# Secure Messaging for Diabetes Management: Content Analysis

**DOI:** 10.2196/40272

**Published:** 2023-03-23

**Authors:** Stephanie A Robinson, Mark Zocchi, Carolyn Purington, Linda Am, Kathryn DeLaughter, Varsha G Vimalananda, Dane Netherton, Arlene S Ash, Timothy P Hogan, Stephanie L Shimada

**Affiliations:** 1 Center for Healthcare Organization and Implementation Research Veterans Affairs Bedford Healthcare System Bedford, MA United States; 2 The Pulmonary Center Boston University School of Medicine Boston, MA United States; 3 The Heller School for Social Policy and Management Brandeis University Waltham, MA United States; 4 Department of Medicine Boston University School of Medicine Boston, MA United States; 5 Department of Population and Quantitative Health Sciences University of Massachusetts Chan Medical School Worcester, MA United States; 6 Department of Population and Data Sciences University of Texas Southwestern Medical Center Dallas, TX United States; 7 Department of Health Law, Policy, and Management Boston University School of Public Health Boston, MA United States

**Keywords:** secure messaging, patient-provider communication, veterans, message content, diabetes, patient portal, T2D, management, support, messaging, glycemic control, communication, engagement, health information, diabetic control, disease outcomes

## Abstract

**Background:**

Secure messaging use is associated with improved diabetes-related outcomes. However, it is less clear how secure messaging supports diabetes management.

**Objective:**

We examined secure message topics between patients and clinical team members in a national sample of veterans with type 2 diabetes to understand use of secure messaging for diabetes management and potential associations with glycemic control.

**Methods:**

We surveyed and analyzed the content of secure messages between 448 US Veterans Health Administration patients with type 2 diabetes and their clinical teams. We also explored the relationship between secure messaging content and glycemic control.

**Results:**

Explicit diabetes-related content was the most frequent topic (72.1% of participants), followed by blood pressure (31.7% of participants). Among diabetes-related conversations, 90.7% of patients discussed medication renewals or refills. More patients with good glycemic control engaged in 1 or more threads about blood pressure compared to those with poor control (37.5% vs 27.2%, *P*=.02). More patients with good glycemic control engaged in 1 more threads intended to share information with their clinical team about an aspect of their diabetes management compared to those with poor control (23.7% vs 12.4%, *P*=.009).

**Conclusions:**

There were few differences in secure messaging topics between patients in good versus poor glycemic control. Those in good control were more likely to engage in informational messages to their team and send messages related to blood pressure. It may be that the specific topic content of the secure messages may not be that important for glycemic control. Simply making it easier for patients to communicate with their clinical teams may be the driving influence between associations previously reported in the literature between secure messaging and positive clinical outcomes in diabetes.

## Introduction

Approximately 29 million Americans have been diagnosed with diabetes [1]. The prevalence of diabetes among veterans is even higher than that in the general population, affecting nearly 25% of Veterans Health Administration patients [2]. Diabetes can lead to other serious problems, including cardiovascular disease, stroke, and loss of limbs [3]. Diabetes is estimated to cost the US health care system US $245 billion annually [2]. Effective management of diabetes is centered around glycemic control, requiring a multifaceted, continuous, and proactive approach that includes patient monitoring and education, lifestyle management, and pharmacologic therapy [4].

Online patient portals such as the Department of Veterans Affairs (VA) My Health***e***Vet, expand patients’ access to health care by facilitating communication with health care teams, such as through secure messaging. Secure messaging offers an electronic exchange of messages between patients and clinical team members. Through secure messaging, patients can inform clinical team members of their health status and progress and receive self-management or remote support. Secure messaging can increase patient engagement and self-management [5], and can further improve patient outcomes [6,7]. Previous work has found improved glycemic control in patients who used secure messaging for 2 or more years compared to nonusers of secure messaging [8]. Moreover, there appears to be a positive association between the frequency and intensity of secure messaging use and glycemic control [9]. However, the exact message content being exchanged between patients and clinical teams, and how that content supports improved diabetes self-management, is less clear.

Previous work has examined the content of patient-team secure messages. In a general patient population, one secure messaging content analysis across two VA primary care clinics documented that medication renewals and refills, scheduling requests, medication issues, and health issues were the most common patient-initiated requests via secure messaging [10]. To our knowledge, only one study to date has examined the content of secure messaging in patients with diabetes and the association with diabetes outcomes [11]. Specifically, Heisey-Grove et al [11] examined a sample of patient-initiated secure messages that were saved to the patients’ charts in one medical center. This analysis found greater improvements in glycemic levels (ie, decreased hemoglobin A_1c_ [HbA_1c_]) when patients engaged in secure messaging where they *sought* information (ie, information-seeking messages) or received messages about procedures or treatments from clinical team members. Conversely, glycemic levels worsened (ie, HbA_1c_ increased) among patients who engaged in secure messages where they *shared* information with clinical team members [11]. The work of Heisey-Grove and colleagues thus offers foundational insights into how patients and their clinical teams leverage secure messaging for diabetes management.

The current study expands on this earlier work by examining a representative selection of patient- and team-initiated secure messages from a national sample of patients with type 2 diabetes. We sought to understand how patients and clinical teams use secure messaging to communicate with one another about diabetes management. Leveraging a mixed methods analytic approach, we adapted and applied a theoretically based taxonomy to analyze the content of secure messages between patients and clinical teams (described in further detail below). We examined differences in secure message content, the role of the secure message initiator (clinical team member role or patient), and associations with glycemic control.

## Methods

### Recruitment

We used data collected for a larger study performed between 2017 and 2020 examining patient portal use in diabetes management [5,12,13]. Participants included veterans with type 2 diabetes who had uncontrolled blood glucose (2012 mean HbA_1c_>8.0% and less than 25% of the year with HbA_1c_<8.0%), as well as *repeated* and *current* use of key portal features. *Repeated use* was defined as having at least two instances of each of the following in 2 out of 3 years between 2013 and 2015: requesting a prescription refill, viewing or downloading health information, and using secure messages. *Current use* included having sent at least four secure messages between January 2016 and June 2017. Surveys about My Health***e***Vet and diabetes management were mailed to a total of 1200 veterans and 448 were returned. Further details regarding the sampling methodology are available elsewhere [5].

### Ethics Considerations

All survey respondents consented for their recent secure messages to be accessed and analyzed. The Institutional Review Board at VA Bedford Healthcare System approved this research (review number 0008).

### Secure Message Thread Coding

Survey respondents’ five most recent secure messaging threads were pulled in 2018. Every message and/or thread was coded based on previously published message coding methods [10], which were inspired by elements of the Taxonomy of Requests by Patients [14]. Binary indicators were created to indicate which threads were initiated by the patient and which were initiated by the clinical team. At the message level, we used binary codes to indicate whether the message was related to a diabetes theme, including blood pressure, cholesterol, physical activity, diet/nutrition, mental health, and specific diabetes-related content. Messages were coded as including diabetes-related content if the message explicitly mentioned diabetes, blood glucose, “sugars,” insulin, endocrine, or other diabetes medications. Messages that were potentially related to diabetes but not explicitly tied to the condition by either the patient or provider were not coded as a diabetes-related message (eg, blood pressure, diet, exercise). Within each message that was specifically coded as diabetes-related, additional subcodes were applied to further describe whether the diabetes content related to one of the following topics: scheduling, referrals, or consults; medication renewals, refills, or other medication-related issues; test results; test issues; health issues; self-reporting; informational; My Health***e***Vet; life issues; complaints; establishing a personal connection; and care coordination.

Each message could be coded for more than one topic (eg, refills and scheduling). The research team met frequently to discuss coding questions, discrepancies, and codebook updates. A codebook was developed prior to reviewing messaging data and was further refined at the beginning of the coding stages. To ensure consistency, two of three research assistants independently coded each message. Interrater reliability (Cohen κ) was calculated iteratively (range 0.57-1.00 across the coding period), and joint coding continued throughout. Coding at the thread level was informed by the codes applied to the content at the message level (ie, if a thread contained one or more messages related to the specified topic, the thread was coded as discussing that topic). Coding at the individual level was informed by the codes applied to the thread level (ie, if a patient or clinical team member engaged in one or more threads related to the specified topic, the message initiator was coded as having discussed that topic over secure messaging).

### Glycemic Control

To determine the percent time in glycemic control in 2018, we calculated the percentage of time in the baseline year a patient was estimated to have sustained control in HbA_1c_ (<8.0%). Time spent in control was calculated based on the Rosendaal method [15,16], using linear interpolation to assign values to each day between successive measurements. After interpolation, we calculated the percentage of time during the year that the interpolated values fell inside the region of control (eg, HbA_1c_<8.0%). Participants whose HbA_1c_ was below 8.0% for more than 75% of 2018 were considered to be in “good” glycemic control. Conversely, participants who spent less than or equal to 75% of the year with an HbA_1c_ level under 8.0% were considered to be in “poor” glycemic control. The *χ*^2^ test was used to examine differences in frequency of content codes between patients in good and poor glycemic control.

## Results

### Patient and Message Characteristics

The patients’ mean age was 67.4 (SD 7.5) years and the mean HbA_1c_ was 8.1% (SD 1.2). The sample was majority male (94%), white (84%), and married (50%). We examined secure messages across 2240 threads (5442 total messages) from 448 patients who responded to our survey. All 5442 messages were coded. Each thread contained a mean of 2.4 secure messages (SD 1.7), ranging from 1 to 24 messages per thread. Among the 2240 coded threads, 87.86% (n=1968) were initiated by the patient (n=1890) or the patient’s caregiver/proxy (n=78). The remaining 12.14% (n=272) of threads were initiated by the clinical team. Among the clinical team–initiated threads, registered nurses initiated the most threads (109/272, 40.1%), followed by physicians (32/272, 11.8%), licensed practical nurses (30/272, 11.0%), other VA staff (28/272, 10.3%), pharmacists (15/272, 5.5%), medical support assistants/health technicians (4/272, 1.5%), other providers (eg, psychologists; 4/272, 1.5%), and nurse practitioners (3/272, 1.1%). The clinical team member was not clearly identifiable in 17.3% (n=47) of the team-initiated threads.

### Message Content

All patients (N=448) initiated at least one thread and 36.4% (n=163) received at least one team-initiated thread (Table 1). Most patients (n=323, 72.1%) engaged in at least one thread pertaining to diabetes-related content, 31.7% (n=142) of the sample engaged in at least one thread about blood pressure, 15.2% (n=68) engaged in at least one thread about cholesterol, 13.8% (n=62) engaged in at least one thread about mental health, 9.4% (n=42) about diet and nutrition, and 4.0% (n=18) about physical activity. Among patients who engaged in at least one thread related to diabetes content (n=323), 90.7% (n=293) engaged in at least one thread related to medication renewals or refills; 81.4% (n=263) discussed scheduling, referrals, or other administrative content; and 63.5% (n=205) discussed medication or equipment issues. Table 2 presents sample secure messages representing each content type as found within the first message in a patient-initiated thread.

Content contained in team-initiated threads was similar to that found in patient-initiated threads. Of the 163 patients that received a team-initiated thread, 74% (n=121) received at least one thread with content related to diabetes. This was followed by blood pressure (n=48, 29%), diet and nutrition (n=19, 12%), physical activity (n=12, 7%), mental health (n=24, 15%), and cholesterol (n=24, 15%). Among the 121 patients who received a team-initiated thread related to diabetes, 88% (n=107) received a thread about medication renewals of refills; 85% (n= 103) about scheduling, referrals, or consults; and 73% (n=88) about medication issues (Table 1). Table 3 presents sample secure messages of each content type as found within the first message in a team-initiated thread.

**Table 1:** Frequency of patients who engaged in at least one secure message thread by thread content (N=448).

Thread content			Patients who initiated a thread (n=448), n (%)	Patients who received a team-initiated thread (n=163), n (%)
**General health topics**				
	Blood pressure		142 (31.7)	48 (29.4)
	Cholesterol		68 (15.2)	14 (14.7)
	Physical activity		18 (4.0)	12 (7.4)
	Diet/nutrition		42 (9.4)	19 (11.7)
	Mental health		62 (13.8)	24 (14.7)
**Diabetes content**				
	Overall		323 (72.1)	121 (74.2)
	**Subtopics^a^**			
		Health issue	96 (29.7)	41 (33.8)
		Medication renew or refill	293 (90.7)	107 (88.4)
		Medication/equipment issue	205 (63.5)	88 (72.7)
		Test issue	79 (24.5)	36 (29.8)
		Test result	81 (25.1)	43 (35.5)
		Self-reporting	67 (20.7)	34 (28.1)
		Scheduling/referral/consult	263 (81.4)	103 (85.1)
		Life issue	19 (5.9)	14 (11.6)
		Technology	36 (11.1)	13 (10.7)
		Complaint	41 (12.7)	12 (9.9)
		Informational	264 (81.7)	26 (21.5)
		Personal connection	5 (1.5)	3 (2.5)
		Care coordination	42 (13.0)	14 (11.6)

^a^Percentages for diabetes-related subtopics are based on the n values for “overall” (ie, n=323 for patients who initiated a thread and n=121 for patients who received a team-initiated thread).

**Table 2:** Example patient-initiated secure messages representative of specific content.

Content		Representative secure message
**Health topics**		
	Blood pressure	My blood pressure has been running about 170 over 90. Is that acceptable? Also am I supposed to come in every three months to have my blood drawn?
	Cholesterol	Good Morning [name], when I was in to see you I forgot to tell you I needed a refill on my Simvastatin 20mg Qty 90 at [pharmacy]. Thank You
	Physical activity	Our last appointment, we discussed the move program. I think I am interested after all. Please put my name in. Also my nifedipine has fallen off my list of refills. Could you refill it for me. Thanks
	Diet/nutrition	I have a question about blood sugar. What can I do to keep my blood sugar from going low in the middle of my night or early morning? I end up eating more than I should and/or the wrong things.
	Mental health	Tried the Buspirone but does not seem to work. Still using 1/2 tablet twice a day (most days) of the [Lorazepam]. Should we talk again? I will be over there first week of Dec.
**Diabetes content**		
	Medication renew or refill	Dear Sir, I need new prescriptions for Fluoxetine, Lisinopril, and Novo fine disposable needles to go with the Solostar insulin pen. Thank you!
	Informational	Made my oncology appointment in [VA^a^ Site] and they referred me to Choice here in [VA CBOC^b^ Site]. That has worked out great. Cancelled my diabetic appointment in [VA Site] and also requested Choice.
	Scheduling/referral/consult	I have an appointment on [date]. If possible I would like to have some time with [team member] to talk about better Insulin management with my pump. Thank you
	Medication/medical equipment issue	My Glucose test meter has stopped functioning. It no longer recognizes the blood sample. Changed the battery but it did no good. What do I need to do to replace it?
	Health issue	Hi, I wanted to let you know that I did not go to my cardiac appt. yesterday as I woke up and it felt like the room was spinning.....I could not drive and had no ride to the VA....the dizziness continued all day into this morning.....it has tempered [sic.] off at this point, but my head still feels a little dizzy at times......it is 19:30 at this time......am feeling better; but wanted to make you aware...
	Test result	Just saw my lab results. A_1c_ was 13.5 June to lowered to 10.4 August. Not scheduled to see DR until December. Still haven’t gotten an appointment for Diabetic Eye exam. Please could I get a direct # to call and schedule in [VA site].
	Test issue	…My blood sugar has really spiked over the last three days…My diet has not changed ie no great ingestion of sweets or heavy carbs…My thinking is that the glucose meter is on the fritz. If so can you order me one through the pharmacy? I have a few issues that I’d like to share with you when we meet. Thanks for your attention.
	Self-reporting readings/measures	I am needing less insulin to maintain low sugars. (See Heath Buddy readings My last A_1c_ was 5.8). I haven't taken any Novolog in several months. I am taking 50 units in the morning of Lantus and 60 at night. How should I proceed with insulin? [Physician name] Kidney doctor has made several Med changes. Thank you.
	Care coordination	I forgot to tell you that I had blood test run here in [State] at the [VA CBOC Site] for my diabetic Dr at [VA Site] he raised insulin to 100 units three times a day and started me on a new pill called saxagliptin HCL…I said I was willing to try anything to get sugar under control. See you when I get back. Thank you
	Complaint	Recently I received 300 1/2 ml insulin needles. Who ordered these? Who is consistently making these errors? If I’m taking 300 units of U-500 insulin 3 times per day; isn't that .6 ml? And the needles are .5 ml. Why does this happen every time?
	Technology	I need a referral to [physician name] as she is no longer in my secure messaging list. Thanks
	Life issue	…I really need to see you by the end of the month because my DOT [Department of Transportation] medical card runs out on [date]. I was given only a 90 day temporary card because of glucose levels in my urine dip. If this isn’t done I will lose my CDL [commercial driver’s license] and will be up the creek…
	Personal connection	N/A^c^

^a^VA: Department of Veterans Affairs.

^b^CBOC: community-based outpatient clinic.

^c^N/A: not applicable; no message within a patient-initiated thread fell under this code.

**Table 3:** Example team-initiated secure messages representative of specific content.

Topic		Representative secure message
**Health topics**		
	Blood pressure	I am unable to assess your blood pressure control on the spironolactone as there are no recent BP reports to review from... I have an appointment available on [date and time options]. Could you come in on Weds. at one of those times so that we could assess your blood pressure appropriately? Thanks!
	Cholesterol	The two medications from the cardiologist have been processed and are on the way to you. They are Metoprolol 100mg twice a day and Atorvastatin 40mg daily. Metoprolol 50mg was discontinued when the cardiologist wrote for the higher dose. Thanks
	Physical activity	Good afternoon [patient name], I hope the weather in [state] is starting to warm up for you… Have you been able to get into the pool to start your exercise?...
	Diet/nutrition	I got a recommendation back from [team member] in regards to your recent secure message. She says that she would be willing to send you to the Endocrine clinic [VA^a^ site] for diabetes management if you would like…You always have the option of working with me but you would need to send in your blood sugar logs after every insulin change. More importantly though, you will need to find a way to eat more consistently so that insulin changes could be made. I could set you up with our Dietician if you would like help in meal planning.
	Mental health	Scheduled you to see [physician name], psychiatrist here at [VA site] for [date and time] for hour visit. Please let us know if this does not work for you. appointment letter will be mailed. Take care.
**Diabetes content**		
	Medication renew or refill	I put in an order for more Accu-chek glucose strips and a renewal for your glucose meter so you are able to check and track of your sugar levels. If you have any questions or concerns feel free to ask. Hope all is well.
	Informational	N/A^b^
	Scheduling/referral/consult	I know you and [team member] (our RN^c^) talked about diabetes case management but thought I would send a clarification. Your ophthalmologist did request diabetes case management through the “main” VA but that consult was discontinued because you are currently a patient here in [VA CBOC^d^ site]. IF you would like to change providers (both primary care and thus a pharmacist case manager), then you would need to make an appointment with a provider at [“main” VA site] for a transfer of care and he/she will then place the consult for pharmacy diabetes management. You can call [phone number] and follow the prompts to make an appointment. Even if you choose to transfer your primary care/pharmacist providers to the “main” VA; you are still welcome to come here to [VA CBOC site] for lab draws; Vitamin B12 shots and Mental Health with [team member]. Those are still all available for you. Please let me know if I can be of any further help; either to facilitate transfer to another provider or to assist in diabetes management.
	Medication/medical equipment issue	I am following up from our visit [date] when we increased your Lantus insulin dose to 68 units daily. What have your morning fasting blood sugars been running since this change? The goal range for fasting blood sugar is between 80 and 130. Let me know how I can assist you with improved diabetes control. Thank you
	Health issue	Unfortunately, we have not been able to reach you by phone. Please be advised that if your blood sugar is over 500 that is a medical emergency and you have to seek care. If you do not have a ride to an ER [emergency room] you will need to call 911. Also consider that being dizzy could be a cardiac issue and also needs to be addressed as an emergency. I hope you did get treatment. Please follow up with us so we know you are ok and can provide the medical care that you require. Also if you are experiencing concerning symptoms call the clinic you will get an advice nurse quicker than sending a secure message.
	Test result	[Team member] has reviewed your recent lab results: 1)Your a_1c_ resulted at 9.3%. This means that your blood sugar has been averaging 219 over the past 3 months. [Team member] strongly recommends that you keep the appointment on [date and time] with the Clinical Pharmacist. This appointment will focus on your diabetes. Please bring your blood sugar meter...
	Test issue	I ordered an A_1c_ for you to get Jan but it is not done. Could you go to the lab on [date] and get it drawn? I appreciate the averages you sent earlier this month, but that does not give me enough information
	Self-reporting	How have your blood sugars been running? Have you been able to get into the pool to start your exercise? If you have a moment would you be able to send me 1-2 weeks worth of blood sugar readings to see if we need to change the doses of your medication. Any issues or concerns we need to address?
	Care coordination	Thank you for providing me with [physician name]’s contact information. As you already signed the release of information form, I will fax it on Monday and await for them to send me your records. I already placed a reminder in my system, if I have not received them within 7 business days, I will contact them directly by phone and have you give them a call as well.
	Complaint	N/A
	Technology	Hi [name]… Hope this works right with your email address!
	Life issue	N/A
	Personal connection	Hi [name] I know about getting older and arthritis, dealing with that myself. To be able to do 7,000 steps is great. Just keep watching the calories and you will be fine. Take Care.

^a^VA: Department of Veterans Affairs.

^b^N/A: not applicable; no message within a team-initiated thread fell under this code.

^c^RN: registered nurse.

^d^CBOC: community-based outpatient clinic.

### Association Between Content and Glycemic Control

From the initial 448 participants, 431 had at least one HbA_1c_ value in 2018. Of the 431, 44.6% (n=192) met our definition of good glycemic control and 55.5% (n=239) met our definition of poor glycemic control in 2018. A significantly larger proportion of patients with good glycemic control (n=72, 37.5%) engaged in at least one thread about blood pressure, compared to those with poor glycemic control (n=65, 27.2%; *P*=.02). Noting this significant difference, we further examined the content among blood pressure–related messages. Similar to the diabetes-related messages, we found that, among patients who engaged in at least one blood pressure–related message (n=142), most patients engaged in messaging related to medication renewals or refills (n=138, 97.2%); followed by scheduling, referrals, or other administrative content (n=108, 76.1%); and medication issues (n=86, 60.6%).

Among those who engaged in at least one thread related to diabetes content (n=312), 43.3% (n=135) were in good glycemic control and 56.7% (n=177) were in poor glycemic control. Significantly more patients in good glycemic control (32/135, 23.7%) engaged in at least one thread related to informing their clinical team about facts relevant to their health care compared to those in poor glycemic control (22/177, 12.4%; *P*=.009). There were no other significant differences related to the proportion of patients in good and poor glycemic control among other health topics of diabetes-related content codes (Table 4).

Among patients in good glycemic control (n=192), 39.9% (n=63) received at least one team-initiated thread. Among patients in poor glycemic control (n=239), 39.8% (n=95) received at least one team-initiated thread. However, this difference was not statistically significant (*P*=.14).

**Table 4:** Proportion of participants who engaged in at least one thread by glycemic control status.

Topic				Poor HbA_1c_^a^ control (n=239), n (%)		Good HbA_1c_ control (n=192), n (%)		*P* value
**Health topics**								
	Blood pressure			65 (27.2)		72 (37.5)		.03
	Cholesterol			35 (14.6)		32 (16.7)		.74
	Mental health			33 (13.8)		25 (13.0)		.98
	Diet/nutrition			24 (10.0)		17 (8.9)		.61
	Physical activity			10 (4.2)		7 (3.6)		.85
**Diabetes-related topics**								
	Overall			177 (74.1)		146 (69.9)		.32
	**Subtopics^b^**							
		Medication renew or refill	161 (91.0)		122 (90)		.86	
		Scheduling/referral/consult	148 (83.6)		105 (78)		.19	
		Medication/equipment issue	117 (66.1)		85 (63)		.57	
		Health issue	60 (33.9)		34 (25)		.10	
		Test result	47 (26.6)		33 (24)		.18	
		Test issue	48 (27.1)		30 (22)		.32	
		Self-reporting	37 (20.9)		27 (20)		.85	
		Complaint	23 (13.0)		17 (13)		.92	
		Technology	22 (12.4)		14 (10)		.57	
		Informational	22 (12.4)		32 (24)		.01	
		Care coordination	22 (12.4)		19 (14)		.18	
		Life issue	11 (6.2)		7 (5)		.15	

^a^HbA_1c_: glycated hemoglobin.

^b^Percentages for diabetes-related subtopics are based on the n values for “overall” (ie, n=177 for patients with poor control and n=146 for patients with good control).

## Discussion

### Principal Findings

We used mixed methods to examine the content in a national sample of patient-team secure messages, and the relationship between content and glycemic control. Approximately one-third of the veterans in our sample received at least one secure message thread that was initiated by a member on their clinical team. The initiator on the clinical team was most frequently a registered nurse. We found that more than half of our sample used secure messaging to discuss diabetes-related content. Among those who used secure messaging to discuss diabetes-related content, more than half discussed medication renewals or refills, scheduling, and medication or equipment issues. The proportion of patients who discussed these topics was similar among patients who received at least one team-initiated secure message. This work is aligned with earlier content analyses [10]. Previous work, which was not specific to patients with diabetes, found that the most common messages are transactional (eg, requests for refills, appointment scheduling, administrative requests), followed by informational (eg, patients providing health measurements or updates on care) or interactional (eg, request for input on medical symptoms or medication issues) [10]. The content of the secure messages was similar in both patient- and team-initiated messages, although patients more frequently initiated a thread to seek a medication renewal, whereas team members most frequently initiated a thread to inquire about medication and medical equipment issues. 

There were few differences in secure messaging content between participants in good and poor glycemic control. One exception was that compared with participants in poor glycemic control, significantly more participants in good glycemic control engaged in secure messaging related to blood pressure. There is a well-established risk inherent in the combination of poor glycemic control and uncontrolled hypertension in patients with type 2 diabetes [17]. As such, blood pressure control is also clinically recommended as an important target for diabetes management [18]. Post hoc analyses revealed that patients who engaged in secure messaging about blood pressure tended to discuss similar topics to those who engaged in secure messaging about diabetes-related content: medication renewals or refills, scheduling, and medication issues. It is possible that patients who were able to obtain good glycemic control are, in general, more active in their disease self-management, and thus leveraging secure messaging to manage other important aspects of their health (ie, blood pressure).

Compared with those in poor glycemic control, significantly more patients in good glycemic control used secure messaging to provide their clinical team with information simply for the sake of keeping them informed about their health status or health care received elsewhere. This contrasts with earlier work, in which Heisey-Grove et al [11], counter to their hypothesis, reported that patients sharing information with clinical team members experienced an increase in HbA_1c_. A plausible explanation as to why our findings differ may be that our definition of information sharing was more constrained. In their analyses, they combined three subtypes of information sharing—clinical updates, self-reporting, and responses to clinician messages—and were unable to detect a statistically significant association for any of the three subtypes with glycemic control. Additionally, these represent both proactive and reactive messages, whereas we focused on proactive messages from patients to inform their teams, which they called “clinical updates.” Patients who engage in this type of proactive communication may be more activated patients, in the sense that they are engaged in managing their own condition and recognize the importance of care coordination. Heisey-Grove et al [11] did find that patients who initiated secure messaging threads experienced HbA_1c_ improvements compared to those who did not. Previous work has found that use of online patient portals and accompanying features such as secure messaging can increase measures related to patient activation [19] and subsequent self-management [5]. This is echoed in the eHealth Enhanced Chronic Care Model [20], which outlines the role that eHealth plays in supporting productive interactions between activated, informed patients and prepared, proactive clinical teams to support chronic disease outcomes. Patient and staff training is crucial to support portal use [21] and may in turn support patient activation and engagement in their care.

The current analysis expands on Heisey-Grove et al’s [11,22] earlier work by examining the association between information sharing and glycemic control with a more complete selection of secure messages (ie, all of a patient’s most recent secure messaging threads, not just those saved to the patient’s chart). We analyzed all secure messages that the patient and team engaged in from the patients’ most recent five secure messaging threads, whereas earlier work only examined secure messages that were selectively saved to the patients’ charts [11]. Secure messages that the clinical team chose to save to patients’ charts may be perceived to have greater clinical relevance, and therefore frequencies of message content and the associations between the content and clinical outcomes may also be biased. For example, at a site that saves only select messages, a team member may be more likely to save a secure message if the patient reported concerning HbA_1c_ levels and the team responded with recommendations for action. They may be less likely to do so if self-reported HbA_1c_ levels were not a cause for concern and no action or change is recommended. Thus, our study adds to the literature by presenting a less biased picture of how veterans living with diabetes and their clinical teams use secure messaging to support diabetes management.

The current analysis also expands on earlier work by examining both patient- and team-initiated secure messaging threads. Compared to a prior study examining the prevalence of team-initiated secure messaging threads in VA [10], we observed more team-initiated threads (10.8% of the threads) in this analysis of 2018 data than we did in 2013 (5.5%). This increase is promising given that clinical team–initiated secure messaging can significantly and positively influence diabetes self-management [5]. By including team-initiated messages, we enhance our understanding of patient-team communication for diabetes via secure messaging. Finally, another novel aspect of the current analysis is that we were able to leverage the United States’ largest integrated health system, the Veterans Health Administration, and examine secure messages from across the nation. Examining the content of secure messages within one medical center may not capture the heterogeneous needs of patients with diabetes. For example, a rural patient with difficulty accessing in-person care may be more reliant on using secure messaging than a patient who lives closer to the medical center [23].

### Limitations

The strengths of the current analysis include the number of secure messages coded, rigorous coding methods, and link to clinical outcomes in a national sample of US veterans with diabetes. There are also several limitations. For one, the sample is predominantly white male US veterans and thus we are limited in the generalizability of these findings. Additionally, we focus on a set of specified topics related to diabetes, although we did not include a specific code about self-management that may have likely led to a change in clinical team action (eg, medication titration) or patient action (eg, decrease a medication dose). Future work should analyze other content areas beyond topic, such as shared decision-making, to perhaps further understand how other content may influence diabetes management. Another avenue for future work would be to explore the impact of training on patient and clinical team use of secure messaging. For example, teaching both patients and clinical teams how to use secure messaging to support disease management may support the adoption and effectiveness of secure messaging for disease management [24].

Another important consideration of this work is that we analyzed the content of secure messaging among patients who were sustained users of secure messaging. As such, our findings may not be generalizable to patients who use secure messaging less frequently or not as recently. Another limitation is that this research examines cross-sectional associations between secure messaging and glycemic control and cannot confirm a causal pathway. For example, patients who share information via secure messaging may be more proactive in their disease self-management, which may lead to improvements in glycemic control. Conversely, patients who share information may be struggling more with controlling their HbA_1c_, which may be associated with worse glycemic control. Future work that examines how secure messaging adoption, and subsequent content of the messages, influences glycemic control will further our understanding of the directionality between content and clinical outcomes.

### Conclusions

This is one of the first studies to perform a content analysis of secure messaging specific to diabetes, and to explore associations between message content and glycemic control. Focusing on a chronic condition where patients’ self-management behaviors drive outcomes allowed for a nuanced exploration of the relationship between secure messaging and health outcomes. In addition to reducing bias in how messages were sampled (ie, coding all patient-team messages as opposed to a sample saved to the patient’s chart), this work adds a complementary perspective to earlier content analyses [11] with supplementary qualitative examples of secure messages. We sought to understand whether secure message content was associated with good or poor glycemic control. It appears that, overall, the specific topic of the secure messaging may not be as clinically important for diabetes management. Rather, the act of engaging (compared to not engaging) in secure messaging may be most influential. Secure messaging makes it easier for patients to communicate with their clinical teams, and this may be the main driver of better clinical outcomes. Clinical teams should be encouraged to communicate, both responsively and proactively, with their patients through secure messaging to support their diabetes management.

## Abbreviations

HbA_1c_: hemoglobin A_1c_
VA: Department of Veterans Affairs

## Data Availability

The US Department of Veterans Affairs (VA) prohibits unauthorized sharing of data. The data used for this study are not permitted to be shared outside the VA firewall without a Data Use Agreement. This limitation is consistent with other studies based on VA data; however, VA data are available to researchers behind the VA firewall with an approved VA study protocol. For more information, please visit the VA Information Resource Center (VIReC) website [25] or contact the VIReC at VIReC@va.gov.
